# Prognostic accuracy of eight triage scores in suspected COVID-19 in an Emergency Department low-income setting: An observational cohort study

**DOI:** 10.1016/j.afjem.2023.12.004

**Published:** 2024-01-26

**Authors:** Carl Marincowitz, Madina Hasan, Yasein Omer, Peter Hodkinson, David McAlpine, Steve Goodacre, Peter A. Bath, Gordon Fuller, Laura Sbaffi, Lee Wallis

**Affiliations:** a Centre for Urgent and Emergency Care Research (CURE), Population Health, School of Medicine and Population Health, University of Sheffield, Regent Court, 30 Regent Street, Sheffield S1 4DA, UK; b Division of Emergency Medicine, Groote Schuur Hospital, University of Cape Town, F51 Old Main Building, Observatory, Cape Town, South Africa; c Information School, University of Sheffield, Regent Court, 211 Portobello St, Sheffield S1 4DP, UK

**Keywords:** Covid-19, Triage, Risk-stratification, Lower- middle- income countries (LMICS) & Emergency Department

## Abstract

**Introduction:**

Previous studies deriving and validating triage scores for patients with suspected COVID-19 in Emergency Department settings have been conducted in high- or middle-income settings. We assessed eight triage scores’ accuracy for death or organ support in patients with suspected COVID-19 in Sudan.

**Methods:**

We conducted an observational cohort study using Covid-19 registry data from eight emergency unit isolation centres in Khartoum State, Sudan. We assessed performance of eight triage scores including: PRIEST, LMIC-PRIEST, NEWS2, TEWS, the WHO algorithm, CRB-65, Quick COVID-19 Severity Index and PMEWS in suspected COVID-19. A composite primary outcome included death, ventilation or ICU admission.

**Results:**

In total 874 (33.84 %, 95 % CI:32.04 % to 35.69 %) of 2,583 patients died, required intubation/non-invasive ventilation or HDU/ICU admission . All risk-stratification scores assessed had worse estimated discrimination in this setting, compared to studies conducted in higher-income settings: C-statistic range for primary outcome: 0.56–0.64. At previously recommended thresholds NEWS2, PRIEST and LMIC-PRIEST had high estimated sensitivities (≥0.95) for the primary outcome. However, the high baseline risk meant that low-risk patients identified at these thresholds still had a between 8 % and 17 % risk of death, ventilation or ICU admission.

**Conclusion:**

None of the triage scores assessed demonstrated sufficient accuracy to be used clinically. This is likely due to differences in the health care system and population (23 % of patients died) compared to higher-income settings in which the scores were developed. Risk-stratification scores developed in this setting are needed to provide the necessary accuracy to aid triage of patients with suspected COVID-19.

**African Relevance**

Uneven vaccination rates alongside less resilient emergency care in settings like Sudan mean that further COVID outbreaks still pose a risk of overwhelming available health services.

Triage tools, including NEWS2, PRIEST and LMIC-PRIEST scores have demonstrated accurate prediction in suspected COVID-19 of death or need for organ support in higher income settings.

In Emergency Unit COVID-19 centres in government hospitals in Sudan there were high rates of serious adverse outcomes (33.84 % death, intubation/non-invasive ventilation in COVID-19 Centre or HDU/ICU admission) during the initial waves of the pandemic.

None of the eight risk-stratification score predicted death or need for organ support with the accuracy needed to help admission decision-making.

Research is needed to develop risk-stratification scores which could be used in this and similar settings.

Alt-text: Unlabelled box

## Introduction

Less resilient health care provision combined with uneven vaccination means that Emergency Department (EDs) in low- and middle- income countries (LMICs), especially in low-resource settings such as Sudan, still may be overwhelmed when there is high COVID-19 prevalence [1,2]. Only around 20 % of the population of Sudan are estimated to be fully vaccinated. Use of Emergency Department (ED) risk-stratification scores can allow patients who need treatment in hospital to be identified quickly. Disposition decisions in LMICs are largely based on clinician gestalt and available clinical experience [3]. Clinical risk-stratification scores may help clinicians with less experience rapidly identify those who need treatment in hospital and increase transparency of decision-making.

The AFEM COVID-19 Mortality Scale (AFEM-CMS) was developed for an Emergency Department setting in the low-resource setting of Sudan and showed good discrimination (C-statistic 0.78), albeit on a small sample size (467 patients), without external validation [4]. Acuity scores developed in high income settings, such as the Pandemic Respiratory Infection Emergency System Triage (PRIEST) score are able to show accurate prediction of a composite outcome of death or organ support in patients with suspected COVID-19 in higher income settings and the PRIEST score is recommended for use in the ED for risk-stratification by the American College of Emergency Physicians to identify patients who may need inpatient treatment [5], [6], [7], [8]. The LMIC-PRIEST score was developed in an ED setting in the Western Cape, South Africa (a middle income country). The LMIC-PRIEST score includes physiological cut-offs based on routine practice in South Africa and includes comorbidities (Heart disease and diabetes), in place of functional status in the PRIEST Score [9]. The accuracy of NEWS 2, PRIEST, LMIC-PRIEST and other clinical risk-stratification scores have not previously been assessed in a low resource setting. If such scores accurately predict serious adverse outcomes based on information available at initial triage they could help identify very low-risk patients who could be discharged immediately in order to help mitigate the risk of hospitals being overwhelmed during periods of increased COVID prevalence.

We aimed to estimate the ability of existing clinical risk-stratification score to predict risk of death or need for respiratory support in those with suspected COVID-19 infection support in Sudan (a low resource setting).

## Methods

We conducted a retrospective observational cohort study that estimated the accuracy of eight clinical risk-stratification scores (PRIEST score, LMIC-PRIEST, Quick Covid Severity Index, TEWS, NEWS2, WHO algorithm, CRB-65 and PMEWS) developed for COVID-19 or other respiratory infections (Supplementary Material 1) [5,8,[10], [11], [12], [13], [14]]. We adhered to STROBE reporting guidelines [15].

### Setting

Our observational retrospective cohort study was conducted in nine government referral hospitals in Khartoum State, Sudan. Study data were derived from an electronic registry of consecutive patients treated for COVID-19 in COVID-19 centres at these nine government hospitals. The study period encompasses the ancestral Wuhan strain, Beta and Delta Waves. Sudan has two types of hospitals: district and referral. District hospitals are in rural and peri‑urban areas and provide less specialist care, while referral hospitals are in urban centres and provide more advanced and specialist care. During the study period patients with suspected COVID-19 pandemic received similar levels of care in centrally managed COVID isolation units in both district and referral hospitals. Screening based on clinical suspicion (COVID-19 testing was very limited in this setting) was introduced during the Covid-19 pandemic in all twenty-one public hospital Emergency Units (EUs) and in nine hospitals a dedicated primary isolation area (Covid-19 centre) was developed to hold, treat and then safely transfer patients with suspected Covid-19 to five secondary Covid-19 treatment hospitals. The already fragile health system in Sudan came under significant pressure, especially during the early phases of the pandemic, with some hospitals closing due to staff sickness. Due to fear infection and knowledge of the pressure the emergency health care system was under, a high degree of patient led population pre-selection occurred, with only the sickest patients attending EUs. No scoring system was used routinely to assess patient acuity and patients were treated in EUs irrespective of severity of illness.

### Data collection

At the nine study sites data were extracted and deidentified from paper records into a secure electronic database stored locally. Physiological parameters and presenting complaints from initial presentation to the Covid-19 centres were collected by clinical staff using electronic forms. These were the first physiological parameters recorded prior to treatment being initiated. Comorbidities and symptoms were recorded as unstructured free text and were extracted using natural language processing with manually checking of data extraction in a subset of patients prior to analysis [16]. If comorbidities were not documented in available records they were assumed not to be present. Recorded observation which appeared implausible were assumed to be recorded in error and changed to missing (Supplementary Material 2). Some physiological variables including temperature and conscious level were not routinely measured or recorded and were excluded from analysis when calculating triage scores.

### Inclusion criteria

All patients aged 16 or more years between 3rd January 2020 and 14th December 2021 who were treated at participating COVID-19 centres. Patients were treated in COVID centres based on a clinical suspicion of COVID-19 infection and all patients were included irrespective of acuity. Patients with incomplete demographic (age or sex) information were excluded.

### Outcome

The primary outcome was a composite of any of: non-invasive ventilation, intubation in Emergency Unit Covid-19 centre, admission to higher dependency care (HDU/ICU) or inpatient death.

There were two separate non-composite secondary outcomes: (1) inpatient death and (2) HDU/ICU admission.

### Analysis

The accuracy of eight clinical risk-stratification scores were estimated for our study outcomes [5,8,[10], [11], [12], [13], [14]]. Discrimination of each score was estimated in terms of the receiver operating characteristic (ROC) curve and the area under the ROC curve (c-statistic). Sensitivity, specificity, positive predictive value (PPV) and negative predictive value (NPV) for each score threshold was estimated at thresholds which have been recommended for use clinically: 0 vs 1+ CRB-65; 0–1 vs 2+ NEWS2; 0–2 vs 3+ PMEWS; 0–4 vs 5+ PRIEST; 0 vs 1 WHO score; TEWS 0–2 vs 2+ Quick COVID Severity Index 0–3 vs 4+ [[13], [17], [18]]. We also estimated accuracy at every score threshold to assess the impact of using different thresholds to guide hospital admissions. We excluded patients on greater than 10 L/min oxygen from the analysis of the Quick COVID Severity Index as this formed part of the outcome in the development study [8]. All analyses were performed in Python 3.8.8. pandas version: 1.4.4 [19].

### Sample size

We used a convenience sample size of data collected on patients with suspected COVID-19 presenting to the nine-participating hospitals 3rd January 2020 and 14th December 2021.

We *a priori* estimated a desired precision of discrimination for the AUC (C-statistic) based on 6000 patients with an event rate of the primary outcome of 5 %. Assuming an AUC of 0.8, based on discrimination of triage scores in other studies [6], this would provide a confidence interval width of 0.06 (95 % CI 0.77 to 0.83). Our study cohort was smaller but had a higher event rate and provided estimated confidence interval widths of a similar size.

Khartoum State Ministry of Health, and the University of Cape Town Human Research Ethics Committee (HREC 450/2020) gave approval for the study. Data were anonymised at each study site prior to being pooled and made available for analysis.*.*

## Results

### Study population

**Fig. 1:**
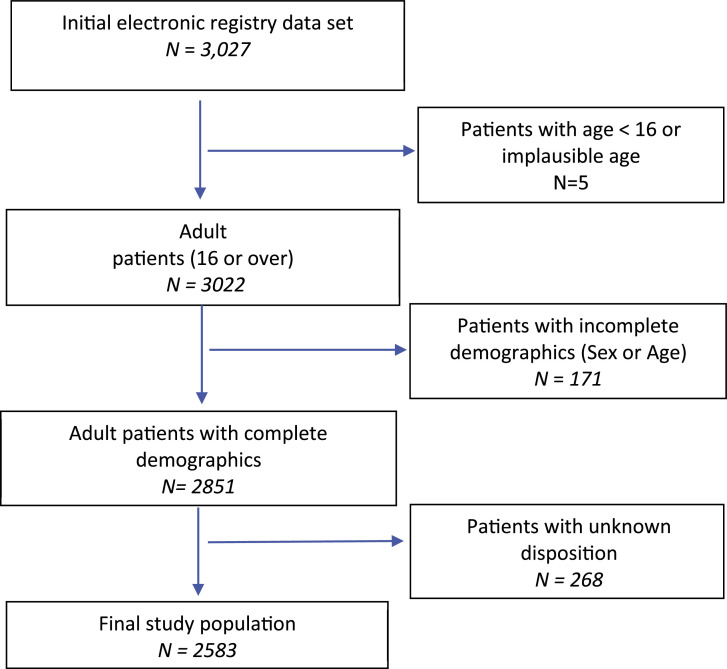
Selection of study cohort (STROBE diagram).

Fig. 1 shows how the study cohort was selected and Table 1 show the demographic and clinical details of the 2583 patients in our study cohort. Overall, 874 (33.84 %, 95 % CI:32.04 % to 35.69 %) died, required intubation/non-invasive ventilation in COVID-19 Centres or HDU/ICU admission, 596 (23.07 %, 95 % CI: 21.49 % to 24.73 %) patients died and 59 (2.28 %, 95 % CI: 1.77 % to 2.93 %) had a HDU/ICU admission. 1248 (48.3 %) of patients had available COVID test results and 77.5 % of tested patients had confirmed infection. There were only around 50 critical care beds available at secondary isolation hospitals. Patients receiving non-invasive ventilation were routinely managed in non-ICU settings and this accounts for small number of patients admitted to HDU/ICU despite 300 patients receiving ventilatory support.

**Table 1:** Study population characteristics.

Characteristic	Statistic/level	Primary outcome(% primary outcome)	No primary outcome (% no primary outcome)	Total (% total population)
	N	874 (33.8 %)	1709 (66.2 %)	2583
Age (years)	Mean (SD)	65.8 (13.7)	62.8 (14.1)	63.8 (14)
	Median (IQR)	68 (59)	65 (55)	65 (55)
	Range	17–99	16–100	16 to 100
Sex	Male	586 (35 %)	1089 (65 %)	1675 (64.8 %)
	Female	288 (31.7 %)	620 (68.3 %)	908 (35.2 %)
Comorbidities	Asthma	2 (33.3 %)	4 (66.7 %)	6 (0.2 %)
	Cardiovascular disease	13 (39.4 %)	20 (60.6 %)	33 (1.3 %)
	Diabetes	14 (28 %)	36 (72 %)	50 (1.9 %)
	Hypertension	122 (36.9 %)	209 (63.1 %)	331 (12.8 %)
	Malignancy	1 (25 %)	3 (75 %)	4 (0.2 %)
	Renal impairment	10 (32.3 %)	21 (67.7 %)	31 (1.2 %)
Symptoms	Cough	35 (30.2 %)	81 (69.8 %)	116 (4.5 %)
Systolic BP (mmHg)	Missing	–	–	797 (30.9 %)
	Mean (SD)	128.7 (26.2)	131.5 (22.5)	130.6 (23.8)
	Median (IQR)	127 (112)	130 (118)	130 (155)
	Range	53–257	60–245	53–257
Heart rate	Missing	–	–	610 (23.6 %)
	Mean (SD)	97.5 (21.4)	91.3 (18.4)	93.5 (19.7)
	Median (IQR)	97 (84)	90 (79)	92 (80)
	Range	28–196	12–155	12–196
Respiratory Rate	Missing	–	–	694 (26.9 %)
	Mean (SD)	33.5 (10.2)	29.8 (9.3)	31 (9.8)
	Median (IQR)	32 (26)	28 (24)	29 (25)
	Range	12–88	10–108	10–108
Short of breath	N	32 (30.2 %)	74 (69.8 %)	106 (4.1 %)
Oxygen Saturation	Missing	–	–	385 (14.9 %)
	Mean (SD)	83.3 (17)	84.3 (14.4)	84 (15.4)
	Median (IQR)	89.5 (77)	88 (80)	88 (79)
	Range	18–100	10–100	10–100
Supplemental Oxygen	Missing	–	–	670 (25.9 %)
	Non-rebreathe Mask	222 (29.1 %)	542 (70.9 %)	764 (29.6 %)
	Room air	150 (22.3 %)	522 (77.7 %)	672 (26 %)
	Nasal Cannula	8 (10.1 %)	71 (89.9 %)	79 (3.1 %)
	Dual flow (e.g. nasal cannula and oxygen mask)	41 (42.3 %)	56 (57.7 %)	97 (3.8 %)
	Simple Face Mask	1 (100 %)	0	1 (0.004 %)
Respiratory Support	Intubated	2 (100 %)	–	2 (0.01 %)
	Non-invasive ventilation	298 (100 %)	–	298 (11.5 %)
Disposition	Transferred	221 (15 %)	1255 (85 %)	1476 (57.1 %)
	Deceased	596 (100 %)	–	596 (23.1 %)
	Discharged	51 (12.3 %)	363 (87.7 %)	414 (16 %)
	Admitted	6 (6.2 %)	91 (93.8 %)	97 (3.8 %)
Swab result	Missing	–	–	1266 (49.0 %)
	Positive	306 (30.8 %)	689 (69.2 %)	995 (38.5 %)
	Negative	81 (32 %)	172 (68 %)	253 (9.8 %)
	No results	34 (49.3 %)	35 (50.7 %)	69 (2.7 %)
Higher Level of care secondary hospital	HDU	35 (100 %)	–	35 (1.3 %)
	ICU	24 (100 %)	–	24 (0.9 %)

Most patients, 1476 (57.1 %), were transferred for treatment in secondary COVID-19 hospitals. A small number, 97 (3.8 %) were admitted to non-COVID-19 hospitals following negative swab results or a sufficient period of isolation. The COVID-19 centres discharged 414 (16 %) patients and 51 (12.3 %) discharged patients had received ventilatory support.

### Triage score performance

**Fig. 2:**
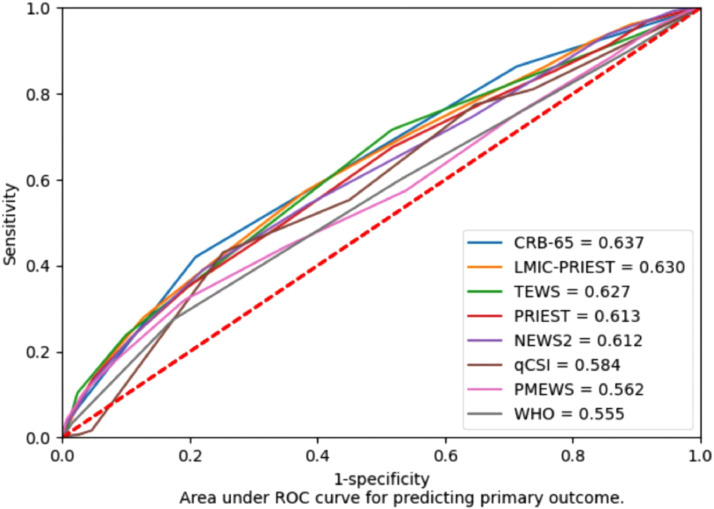
Discrimination of triage scores (receiver operating characteristic curves).

All triage scores assessed had poor estimated discrimination (C-statistic range 0.56–0.64) for the primary outcome (Fig. 2). Estimated discrimination of the risk-stratification scores was higher for the secondary outcome of death (C-statistic range 0.59–0.69) (Supplementary Material 3) and lower for secondary outcome of HDU/ICU admission (C-statistic range 0.46–0.65) (Supplementary Material 4).

Table 2 presents the estimated sensitivity, specificity, positive and negative predictive values at recommended score thresholds for use clinically for the primary composite outcome. Supplementary Material 5 presents estimates for sensitivity, specificity, positive and negative predictive values at every possible score threshold. Tables 3 and 4 present equivalent estimates at recommend score thresholds to Table 2 for the secondary outcomes (1) death and (2) HDU/ICU admission.

**Table 2:** Accuracy triages scores primary outcome (95 % confidence interval).

Score	N*	Outcome (%)	C-statistic	Threshold	N (%) above threshold	Sensitivity	Specificity	PPV	NPV
CRB-65	1659	32.7 (30.5, 35.0)	0.64 (0.61, 0.67)	>0	1264 (76.2 %)	0.86 (0.83, 0.89)	0.29 (0.26, 0.32)	0.37 (0.34, 0.40)	0.81 (0.77, 0.85)
NEWS2	2019	35.2 (33.1, 37.3)	0.61 (0.59, 0.64)	>1	1959 (97 %)	0.99 (0.98, 1)	0.04 (0.03, 0.05)	0.36 (0.34, 0.38)	0.92 (0.82, 0.97)
PMEWS	2254	35.3 (33.3, 37.3)	0.56 (0.54, 0.59)	>2	1965 (87.2 %)	0.89 (0.86, 0.91)	0.14 (0.12, 0.16)	0.36 (0.34, 0.38)	0.69 (0.64, 0.75)
LMIC-PRIEST	2583	33.8 (32, 35.7)	0.63 (0.61, 0.65)	>3	2366 (91.6 %)	0.96 (0.95, 0.97)	0.11 (0.09, 0.12)	0.36 (0.34, 0.38)	0.85 (0.79, 0.89)
PRIEST	2358	35.2 (33.3, 37.2)	0.61 (0.59, 0.64)	>4	2204 (93.5 %)	0.97 (0.95, 0.98)	0.08 (0.07, 0.10)	0.37 (0.35, 0.39)	0.83 (0.76, 0.89)
WHO	2243	35.3 (33.3, 37.3)	0.56 (0.53, 0.58)	>0	2002 (89.3 %)	0.9 (0.88, 0.92)	0.11 (0.01, 0.13)	0.36 (0.34, 0.38)	0.67 (0.61, 0.73)
TEWS	1632	32.6 (30.3, 34.9)	0.63 (0.60, 0.66)	>2	949 (58.1 %)	0.72 (0.68, 0.75)	0.48 (0.45, 0.51)	0.4 (0.37, 0.43)	0.78 (0.75, 0.81)
Quick⁎⁎ COVID	1422	21.9 (19.7, 24.1)	0.58 (0.55, 0.62)	>3	414 (29.1 %)	0.43 (0.38, 0.49)	0.75 (0.72, 0.77)	0.32 (0.28, 0.37)	0.82 (0.80, 0.85)

⁎ Patients excluded if had <3 predictor parameters.

⁎⁎ Patients on greater than 10 L/min oxygen were excluded from analysis.

**Table 3:** Accuracy triages scores death (95 % confidence interval).

Score	N*	Outcome (%)	C-statistic	Threshold	N (%) above threshold	Sensitivity	Specificity	PPV	NPV
CRB-65	1659	22.9 (20.9, 25.0)	0.69 (0.66, 0.71)	>0	1264 (76.2 %)	0.91 (0.88, 0.94)	0.28 (0.26, 0.31)	0.27 (0.25, 0.3)	0.91 (0.88, 0.94)
NEWS2	2019	24.4 (22.5, 26.3)	0.65 (0.63, 0.68)	>1	1959 (97 %)	0.99 (0.98, 1)	0.04 (0.03, 0.05)	0.25 (0.23, 0.27)	0.93 (0.84, 0.98)
PMEWS	2254	35.3 (33.3, 37.3)	0.66 (0.64, 0.69)	>2	1965 (87.2 %)	0.94 (0.92, 0.96)	0.15 (0.13, 0.17)	0.26 (0.24, 0.28)	0.89 (0.84, 0.92)
LMIC-PRIEST	2583	23.1 (21.5, 24.7)	0.67 (0.64, 0.69)	>3	2366 (91.6 %)	0.96 (0.94, 0.97)	0.1 (0.08, 0.11)	0.24 (0.22, 0.26)	0.88 (0.82, 0.92)
PRIEST	2358	23.5 (21.8, 25.3)	0.67 (0.64, 0.70)	>4	2204 (93.5 %)	0.98 (0.96, 0.99)	0.08 (0.07, 0.09)	0.25 (0.23, 0.27)	0.92 (0.86, 0.95)
WHO	2243	24.1 (22.4, 25.9)	0.64 (0.61, 0.66)	>0	2002 (89.3 %)	0.95 (0.93, 0.97)	0.13 (0.11, 0.14)	0.26 (0.24, 0.28)	0.89 (0.84, 0.93)
TEWS	1632	22.9 (20.8, 25.0)	0.67 (0.64, 0.70)	>2	949 (58.1 %)	0.78 (0.73, 0.82)	0.48 (0.45, 0.51)	0.31 (0.28, 0.34)	0.88 (0.85, 0.90)
Quick⁎⁎ COVID	1422	21.3 (19.2, 23.5)	0.59 (0.55, 0.62)	>3	414 (29.1 %)	0.44 (0.38, 0.49)	0.75 (0.72, 0.77)	0.32 (0.27, 0.37)	0.83 (0.81, 0.85)

⁎ Patients excluded if had <3 predictor parameters.

⁎⁎ Patients on greater than 10 L/min oxygen were excluded from analysis.

**Table 4:** Accuracy triages scores HDU/ICU admission (95 % confidence interval).

Score	N*	Outcome (%)	C-statistic	Threshold	N (%) above threshold	Sensitivity	Specificity	PPV	NPV
CRB-65	1659	1.9 (1.3, 2.7)	0.63 (0.55, 0.71)	>0	1264 (76.2 %)	0.94 (0.79, 0.99)	0.24 (0.22, 0.26)	0.02 (0.02, 0.03)	1 (0.98, 1)
NEWS2	2019	2.3 (1.7, 3.0)	0.58 (0.51, 0.66)	>1	1959 (97.0 %)	0.98 (0.89, 1)	0.03 (0.02, 0.04)	0.02 (0.02, 0.03)	0.98 (0.91, 1.0)
PMEWS	2254	2.3 (1.7, 3.0)	0.56 (0.49, 0.64)	>2	1965 (87.2 %)	0.96 (0.87, 1)	0.13 (0.12, 0.15)	0.03 (0.02, 0.03)	0.99 (0.98, 1)
LMIC-PRIEST	2583	2.3 (1.7, 2.9)	0.59 (0.51, 0.66)	>3	2366 (91.6 %)	0.97 (0.88, 1)	0.09 (0.08, 0.1)	0.02 (0.02, 0.03)	0.99 (0.97, 1)
PRIEST	2358	2.3 (1.8, 3)	0.57 (0.49, 0.64)	>4	2204 (93.5 %)	0.96 (0.88, 1)	0.07 (0.06, 0.08)	0.02 (0.02, 0.03)	0.99 (0.95, 1)
WHO	2243	2.3 (1.7, 3)	0.58 (0.51, 0.65)	>0	2002 (89.3 %)	0.96 (0.87, 1)	0.11 (0.1, 0.12)	0.02 (0.02, 0.03)	0.992 (0.97, 1)
TEWS	1632	1.8 (1.2, 2.6)	0.65 (0.56, 0.73)	>2	949 (58.1 %)	0.83 (0.65, 0.94)	0.42 (0.40, 0.45)	0.03 (0.02, 0.04)	0.99 (0.98, 1)
Quick⁎⁎ COVID	1422	1.1 (0.6, 1.8)	0.46 (0.31, 0.62)	>3	414 (29.1 %)	0.31 (0.11, 0.59)	0.71 (0.69, 0.73)	0.01 (0.004, 0.03))	0.99 (0.98, 1)

⁎ Patients excluded if had <3 predictor parameters.

⁎⁎ Patients on greater than 10 L/min oxygen were excluded from analysis.

At previously recommended thresholds three risk-stratification scores (NEWS2, PRIEST and LMIC-PRIEST) had high estimated sensitivities (≥0.95) for the primary outcome (Table 2). However, the baseline risk of 33.8 % meant that low-risk patients identified at these thresholds had an 8 % and 17 % risk of the primary outcome. High sensitivities were achieved at the expense of poor specificity, with very few patients below the previously recommended thresholds.

## Discussion

This study assessed for the first time the accuracy of eight clinical triage scores in the low resource setting of Sudanese COVID-19-units. All risk-stratification scores assessed had worse estimated discrimination compared to higher-income-settings in which the scores were developed and validated [5,8,[10], [11], [12], [13], [14]]. In the study setting there were high rates of death (23.1 %) and the primary composite outcome (33.8 %). This far exceeds the rates estimated in the original PRIEST and LMIC-PRIEST cohorts and represents a much sicker cohort of patients [5,9]. Due to fears of hospital acquired infection and restrictions on available emergency care due to the pandemic, patients only attended hospital as a last resort. This preselection of patients contributed to the high baseline risk, which meant that although previously recommended thresholds for risk-stratification scores to aid discharge decision-making could achieve high (>0.95, PRIEST and LMIC-PRIEST scores) sensitivities for the primary outcome, low-risk patients identified by these thresholds still had an around 15 % risk of the primary outcome. In this context, triage tools intended to rule out severe disease may not be of clinical use.

### Comparison to previous literature

Few studies have been conducted assessing outcomes or potential triage methods for patients with suspected COVID-19 in Sudan. In a study conducted during the first wave of COVID-19 case fatality rates varied between 40 % and 70 % from April to September 2020 in government referral hospitals [4]. In a study conducted COVID-19 treatment hospitals from May 2020 to May 2021 in Gezira State, Sudan, 61.2 % of admitted patients were reported to have received either non-invasive or invasive ventilatory support [20]. The AFEM COVID-19 Mortality Scale (AFEM-CMS) was developed using data from 467 patients with confirmed COVID-19 who presented at two government referral hospitals [4]. The mortality scale achieved a C-statistic of 0.78 (95 % CI:0.737–0.813) on internal validation when predicting death, which compares to estimated c-statistics of 0.59 to 0.69 for the triage scores assessed in this study. However, a key predictor in the AFEM-CMS is conscious level which we found to be poorly collected and recorded within the available emergency COVID-19 centre dataset.

Within higher resource settings of the UK and South Africa the triage scores assessed consistently had higher estimated discrimination for the primary outcome, C-statistics: UK 0.61 to 0.8 and Western Cape, South Africa 0.7 to 0.82 [6,21]. This compared to estimated C-statistics of 0.56 to 0.64 in our Sudanese study cohort. This could be partly explained by some predictors (conscious level and temperature) included in some triage scores being unavailable. However, the strongest predictors of adverse outcomes were previously found to be need for supplemental oxygen, saturations and respiratory rate, all of which were available [5,9]. More plausibly, differences in triage score performance may reflect differences between the Sudanese study population and population characteristics in higher-income settings. The Sudanese population had a high adverse event rate (33.8 %), only 26 % of patients did not require supplemental oxygen on presentation, mean presenting saturations were 84 % and mean initial respiratory rate was 31. In a UK ED setting the prevalence of adverse outcomes was 22.1 %, 68.4 % of patients did not initially require supplemental oxygen, mean presenting saturations were 94.9 % and mean respiratory rate was 23.1. In a South African ED setting 3.45 % of patients experienced the primary outcome, 91.9 % of patients did not require supplemental oxygen on presentation, mean presenting saturations were 96.1 % and mean respiratory rate was 18.6. The differences in the prevalence of outcome and predictors between this Sudanese validation cohort and the development cohorts may have contributed to spectrum effects and the differences in estimated performance [22].

## Limitations

This study appears to be the largest multi-centre cohort study of patients with suspected COVID-19 being treating in the emergency care setting of Sudan. However, data collection was reliant on clinical staff extracting clinical predictors and outcomes from available clinical notes. Although intended to be a consecutive cohort of patients, there may be risk of bias from identification and inclusion of patients in the registry. Information bias may result from predictor misclassification or erroneous data, particularly for recording of comorbidities. The effect of missing data was not assessed using sensitivity analyses. Some predictors used in the triage scores assessed, including conscious level and temperature, were not routinely measured or recorded which may have affected the estimated accuracy. Outcomes including death were only measured if they occurred in hospital and therefore, for the small number of patients discharged from COVID-19 centres, death in the community may have been missed. Additionally, it was not possible to assess prediction of outcomes within specific time periods. The study population is based on those treated in COVID-19 centres due to a clinical suspicion of COVID-19 infection. Clinical suspicion was based on available clinical guidelines and stage of the pandemic. However, 75.6 % of available swab results were positive for COVID-19.

### Implications

Both emergency medicine and formal triage of patient acuity on arrival to emergency care are still being developed in Sudan with no consistent triage system used in our study setting [23]. Reconfiguration of Emergency Department services in order to isolate patients with suspected COVID-19 further disrupted care pathways of patients and our study population was selected from patients treated in COVID-19 centres set up in participating hospitals emergency units. Within this setting none of the triage scores assessed showed sufficient accuracy to be used to select patients who could be safely rapidly discharged following initial assessment. Moreover, some components of the triage scores assessed including conscious level and temperature were not routinely recorded which may affect the feasibility of implementing such scores in this setting. The high adverse event rate and physiological indicators of respiratory failure in the study cohort shows a large degree of population selection had already occurred before patients attended the COVID-19 centres with almost all patients requiring further inpatient treatment (only 16 % of patients were discharged).

Whether the assessed tools would have any clinical use in a population of less seriously ill patients in Sudan (potentially in a community or prehospital setting) requires further research. However, our study indicates the assessed clinical scores should not be adopted in the setting of Emergency Units in Sudan. Within this setting where intensive care capacity is limited, research is needed to develop accurate risk-prediction, and help determine which patients are most likely to benefit from respiratory and other forms of organ support.

## Conclusion

Patients treated in emergency COVID-19 centres in Sudan had high rates of death and need for respiratory support compared to those presenting to Emergency Departments in higher-income settings. No triage score assessed was accurate enough to be used clinically. Further research is needed to identify predictors of adverse outcomes and develop risk-stratification scores which can be used to clinically risk-stratify patients in this setting.

## Dissemination of results

In addition to promotion of the results through study and funder website, findings will be presented informally to clinicians at data collection sites.

## Authors Contribution

Authors contributed as follow to the conception or design of the work; the acquisition, analysis, or interpretation of data for the work; and drafting the work or revising it critically for important intellectual content: CM 20%, MH 15%, YO 15%, PH 7%, DM 7%, SG 7%, PB 7%, GF 7%, LS 7% and LW 7%. All authors approved the version to be published and agreed to be accountable for all aspects of the work.

## Declaration of Competing Interest

The authors declared no conflicts of interest.

## Appendix Supplementary materials

Image, application 1
